# A Polyprotein-Expressing Salmonid Alphavirus Replicon Induces Modest Protection in Atlantic Salmon (*Salmo Salar*) Against Infectious Pancreatic Necrosis

**DOI:** 10.3390/v7010252

**Published:** 2015-01-19

**Authors:** Azila Abdullah, Christel M. Olsen, Kjartan Hodneland, Espen Rimstad

**Affiliations:** 1 Department of Food Safety and Infection Biology, Faculty of Veterinary Medicine and Biosciences, Norwegian University of Life Sciences, P.O. Box 8146 Dep, 0033 Oslo, Norway; E-Mails: azila.binti.abdullah@nmbu.no (A.A.); christel.moraeus.olsen@nmbu.no (C.M.O.); 2 MSD Animal Health Norway, Thormøhlensgate 55, N-5008 Bergen, Norway; E-Mail: kjartan.hodneland@merck.com

**Keywords:** infectious pancreatic necrosis, Atlantic salmon, vaccination, alphavirus replicon

## Abstract

Vaccination is an important strategy for the control and prevention of infectious pancreatic necrosis (IPN) in farmed Atlantic salmon (*Salmo salar*) in the post-smolt stage in sea-water. In this study, a heterologous gene expression system, based on a replicon construct of salmonid alphavirus (SAV), was used for *in vitro* and *in vivo* expression of IPN virus proteins. The large open reading frame of segment A, encoding the polyprotein NH2-pVP2-VP4-VP3-COOH, as well as pVP2, were cloned and expressed by the SAV replicon in Chinook salmon embryo cells (CHSE-214) and epithelioma papulosum cyprini (EPC) cells. The replicon constructs pSAV/polyprotein (pSAV/PP) and pSAV/pVP2 were used to immunize Atlantic salmon (*Salmo salar*) by a single intramuscular injection and tested in a subsequent IPN virus (IPNV) challenge trial. A low to moderate protection against IPN was observed in fish immunized with the replicon vaccine that encoded the pSAV/PP, while the pSAV/pVP2 construct was not found to induce protection.

## 1. Introduction

Infectious pancreatic necrosis virus (IPNV) is the prototype virus of the genus *Aquabirnavirus* in the family *Birnaviridae*. It has a bi-segmented dsRNA genome that is contained in a single shelled icosahedral capsid. Aquabirnaviruses have a global distribution and have been isolated from many different fish families. Viruses are often isolated without particular disease association. Serologically, the aquabirnaviruses can be classified into three serogroups, A-C, and can be further divided into serotypes based on reciprocal neutralization assays [1,2]. Serogroup A viruses can be divided into seven genogroups, based upon variations of the VP2 gene [3]. Aquabirnaviruses may cause diseases, such as infectious pancreatic necrosis (IPN), in farmed salmonids, and nephroblastoma and branchionephritis in eel [4], but often the infection remain subclinical. IPNV was the first virus to be isolated from fish [5], and, in Norway, the disease was reported and virus was isolated from rainbow trout (*Oncorhynchus mykiss*) in 1975 [6]. IPN in salmonid fish is characterized by hyperpigmentation, exophthalmia and petechial hemorrhage on the ventral surface and in the pyloric area, as well as histopathological findings, such as focal necrosis in kidney and pancreas. Clinical, macro- and histopathological findings, in combination with immune staining of viral antigens, make up the basis for laboratory disease diagnosis [7].

The large open reading frame (ORF) of the genome segment A encodes the polyprotein of NH2-pVP2-VP4-VP3-COOH. The polyprotein is cleaved during translation by the non-structural protease VP4 [8], resulting in two structural peptides, pVP2 and VP3. pVP2 is further trimmed by host cell proteases in its carboxy terminus to mature VP2 [8,9,10]. VP2 is the major capsid protein and determinative for the humoral immune response of the fish [11] and important for the virulence of IPNV in Atlantic salmon [12]. VP3 is the internal RNA binding protein, forms the scaffold for assembly of the capsid [9], and has been found to interact with VP2, VP1, and dsRNA [13,14]. VP3, in combination with VP2, assembles virus-like particles (VLP) with a diameter size around 60 nm [15,16].

Improved management, such as detection and removal of IPNV-carrier brood fish and the use of influx water from wells can alleviate IPN in the fresh water phase. Transmission through water and environment cannot easily be controlled in the sea-water phase due to the use of open net structures. IPN outbreaks are usually associated with stress in sea-water-reared post-smolts and grower fish [17], with mortality ranging from 3% to 30% [12,18,19]. The current commercially available vaccines for Atlantic salmon (*Salmo salar*) aim to give protection in the grower stage in sea-water, and are administrated as an oil-adjuvanted intra peritoneal (i.p.) injection containing inactivated virus particles from cell cultures or recombinantly produced capsid protein [20,21]. Many of the IPN vaccination trial attempts have been inconclusive due to lack of consistency in the challenge model [22,23]. There is information regarding vaccine efficacy under field conditions [24,25]. In a comparative test for IPN vaccines in Atlantic salmon, where the vaccines contained either whole virus antigens, whole virus antigens entrapped in nanoparticles, *E. coli* expressed subunit antigens fused with putative translocating domains of Pseudomonas aeruginosa exotoxin, or plasmid DNA encoding segment A, there were moderate differences in performance between the antigen groups, but the whole virus antigen vaccines conferred highest protection [26].

Alphavirus replicon vectors, where the non-structural genes are retained and viral structural protein genes are exchanged with a gene of interest (GOI), have been developed from several different mammalian alphaviruses, such as Semliki forest virus (SFV), Sindbis virus, or Venezuelan equine encephalitis virus (VEE) [27]. These vectors utilize the sub-genomic alphavirus promoter between the non-structural and structural ORFs to provide high expression of the GOI [28,29]. Furthermore, the viral intermediate products activate the innate immune response [30,31]. A replicon system, based on salmonid alphavirus subtype 3 (SAV3) isolated from farmed Atlantic salmon (*Salmo salar*), has been developed [32,33] and found to induce efficient protection against infectious salmon anemia (ISA) and pancreas disease (PD) in challenge experiments [34,35]. The present study was conducted to study a SAV3 replicon driven expression of IPNV segment A polyprotein and VP2 *in vitro* and *in vivo*.

## 2. Materials and Methods

### 2.1. Cells and Viruses

Chinook salmon embryo cells (CHSE-214, ATCC CRL-1681) and epithelioma papulosum cyprini (EPC, ATCC CRL-2872) cells were maintained in Leibovitz’s-15 (L-15, Life technologies, Paisley, Scotland) supplemented with 10% fetal bovine serum (FBS, PAA Laboratories,Pasching, Austria), 2 mM L-glutamine, 0.04 mM β-mercaptoethanol and gentamycin (50 μg/mL) for propagation of cells, while L-15 medium containing 2% fetal calf serum (FCS) was used for virus production. Both cell lines were maintained at 20 °C.

The IPNV serotype Sp was used throughout the study [23]. The virus was purified as described before [36]. Briefly, virus was cultivated in CHSE-214 cells with L-15 medium containing 2% FBS at 15 °C for 3–4 days or until the CPE was extensive. The flasks were freeze-thawed 3 times and the cell debris was spun down at 3300 × *g* for 2 h (Sorvall, Thermo Fisher Scientific, Waltham, MA USA). After centrifugation, the pellet was suspended in 1.8 mL TNE buffer (0.01 M Tris-HCl, 0.1 M NaCl, 0.001 M EDTA, pH 7.2) and overlaid a cesium chloride gradient prepared at 20%, 30% and 40% and centrifuged at 16,000 × *g* for 18 h (SW40 rotor). The gradient was harvested into 1 mL fractions and density was measured using a refractometer (Zeiss, Jena, Germany) and the virus fraction was collected at 1.336 g/cm^3^. The virus fraction was overlaid on 20% sucrose solution in TNE buffer and centrifuged at 100,000 × *g* for 1 h. The pellet was suspended in 0.5 mL PBS (0.14 M NaC1, 2.7 mM KC1, 0.88 mM KH_2_PO_4_, 7.6 mM Na_2_HPO_4_, pH 7.2) and the protein concentration was quantified using a NanoDrop ND-1000 spectrophotometer (NanoDrop Technologies, Wilmington, DE, USA). Purified virus was kept at 4 °C for future use.

### 2.2. Construction of DNA-Layered SAV Replicon Vectors

**Figure 1:**
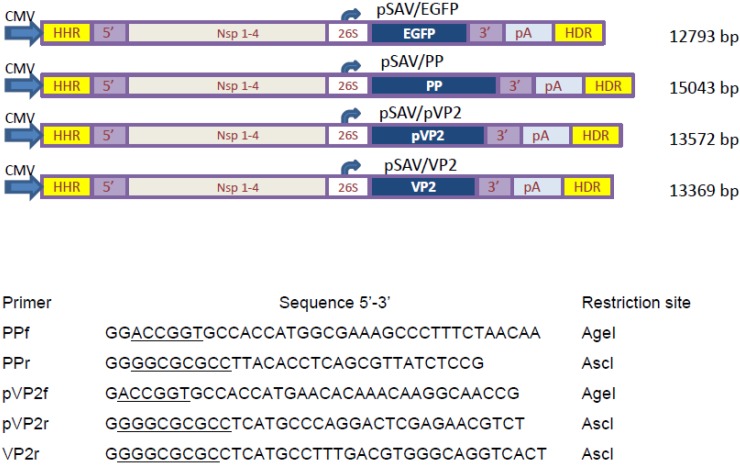
Schematic representation of the DNA-layered, SAV-based replicon vectors pSAV/EGFP, pSAV/PP, pSAV/pVP2 and pSAV/VP2, and listed primers used for construction. CMV immediate early promoter (CMV); Hammerhead ribozyme (HHR); Hepatitis delta virus ribozyme (HDR); 5’ untranslated region (5’); nonstructural protein genes of SAV-3 (Nsp 1-4); subgenomic promoter (26S); enhanced green fluorescent protein (EGFP); polyprotein of IPNV (pSAV/PP); pVP2 precursor of VP2 protein (pSAV/pVP2); VP2 protein (pSAV/pVP2). Restriction enzyme sites are underlined.

The SAV replicon was cloned in a pCI mammalian expression vector backbone (Promega; Madison, WI, USA), as described earlier [35]. The cloning site for the GOI is flanked by *AgeI* and *AscI* restriction enzyme sites. For identification purposes, due to the ubiquitous nature of IPNV, silent mutations in the large ORF of IPNV segment A were introduced at positions 18, 218, 784 and 796 using RT-PCR QuickChange Multi-site-directed mutagenesis Kit (Agilent Technologies, Santa Clara, CA, USA). The gene sequences encoding the large ORF of segment A, the pVP2 or VP2 were cloned into the replicon vector at the GOI site (primer sequences are displayed in Figure 1). The PCR products were run in 1.6% agarose gels, excised and purified with Zymoclean™ Gel DNA Recovery kit (Zymo Research, Irvine, CA, USA) as recommended by the manufacturer. The DNA fragments were cloned into the *Age1* and *Asc1* sites using Pfu UltraII fusion HS DNA polymerase (Stratagene, Agilent technologies) following the manufacturer’s instructions.

The ligated products were transformed into XL10 gold ultracompetent cells, and all inserts were confirmed by restriction enzyme analysis and Sanger sequencing (GATC-Biotech AG, Konstanz, Germany). The replicon plasmids were purified using NucleoBond^®^ Xtra Maxi-EF (Macherey-Nagel, Düren, Germany). The replicons were named pSAV/PP, pSAV/pVP2 and pSAV/VP2 and were kept at −80 °C until further use (Figure 1).

### 2.3. Expression of Recombinant IPNV Proteins in Cell Culture

CHSE-214 and EPC cells were transfected by electroporation (Amaxa-T-20 program, Lonza, Basel, Switzerland) and Ingenio transfection reagents (Mirus, Madison, WI, USA) using approximately 2–4 million cells and 2 µg of each plasmid pSAV/PP, pSAV/pVP2 and pSAV/VP2 per transfection. The plasmids pSAV/EGFP and pMAX/EGFP, both expressing green fluorescent protein was used as control for transfection efficiency. The transfected cells were subsequently incubated in either T-25 flask for downstream Western blot analysis, or distributed (2.5 × 10^5^) (CHSE-214) onto glass coverslips in 24-well culture dish for immunofluorescence staining. The cells were incubated in L-15 medium with 10% FCS at 20 °C for 24 h, followed by changing to fresh media with 2% FCS before transfer to 15 °C for further 4, 6, or 8 days. The experiments were repeated twice.

### 2.4. Immunofluorescence Staining

At 6 or 8 days post transfection (dpt), cells were fixed with 80% cold acetone, washed with PBS and blocked with 10% FCS in PBS (pH 7.4) for 30 min. Primary antibodies were either polyclonal rabbit anti-IPNV (1:5000) [7], or anti-VP2 or anti-VP3 MAbs (both 1:5000) (MAb-Austral Biologicals). Secondary antibodies were Alexa Fluor 594-conjugated goat anti-rabbit IgG and Alexa Fluor 488-conjugated goat anti-mouse IgG Antibody (Molecular Probes, Life technologies, Paisley, Scotland), both were diluted 1:1000. All primary and secondary antibodies were diluted in 1% FCS prepared in PBS. After incubation for 1 h at room temperature with primary antibodies, the cells were washed with PBS for 3 × 5 min and incubated with secondary antibodies for 30 min. DNA and nuclei were counter-stained with Hoechst 33,342 (1 μg/mL). pSAV/EGFP expression was monitored daily. Finally, cells were washed, dried, and mounted with coverslips (Fluoroshield™ Sigma-Aldrich, St. Louis, MO, USA) before viewed under a fluorescence light microscope (Olympus IX81, Center Valley, PA, USA) supplied with cell F software.

### 2.5. Western Blotting

Expression of IPNV proteins in cell cultures was also verified by Western blots of lysate of transfected cells or cell culture medium. The cells or cell culture medium were dissolved in lysis buffer (50 mM Tris-HCl, pH 7.5, 150 mM NaCl, 2 mM EDTA, 1% Triton X-100) and the proteins were separated on a Criterion XT Bis-Tris gel 4%–12% (Bio-Rad; Hercules, CA, USA), with XT-MOPS as running buffer and blotted onto a polyvinylidene difluoride (PVDF) membrane (Bio-Rad) following the Criterion™ Precast Gel system protocol (Bio-Rad). The blot was incubated with polyclonal anti-IPNV (1:5000) or anti-VP3 antibody (1:1000) overnight followed by HRP conjugated anti-rabbit IgG antibody and anti-mouse IgG, respectively, for 2.5 h, and 5% non-fat milk in PBS-SIFF (PBS in 0.1% Tween-20) were used as blocking solution. The membranes were then incubated with substrate from ECL Plus™ Western Blotting (GE HealthCare, Cleveland, OH, USA) for 5 min and detected with ChemiDoc XRS (Bio-Rad).

### 2.6. Vaccination and Experimental Challenge

A cohabitation challenge was performed at VESO Vikan aquatic research facility, Vikan, Norway. The experiment was approved by the Norwegian Animal Research Authority. The trial was performed using unvaccinated IPN-sensitive Atlantic salmon smolts, confirmed free of known salmon pathogens and with average weight of 38 g. The fish were acclimatized for 2 weeks, and kept in sea-water at 12 °C throughout the experiment, fed according to standard procedures, and anesthetized by bath immersion (2–5 min) in benzocaine chloride (0.5 g/10 L water) (Apotekproduksjon AS; Oslo, Norway) before handling. The fish were divided into 4 groups of 35 fish, marked by passive integrated transponder (PIT) tag and immunized by intramuscular injection of 10 µg/50 µL pSAV/PP, pSAV/pVP2 or pSAV/EGFP. Control fish were injected intraperitoneally with 50 µL PBS. IPNV injected shedder fish (N = 53), labeled by adipose fin clipping, were introduced after 40 days. The fish were observed daily and mortality was recorded. Fish were killed using concentrated benzocaine chloride (1 g/5 L water) for 5 min.

Approximately 10% of the dead fish were examined for bacterial infections, and a representative selection of kidney from dead fish after challenge (N = 20) were sampled and tested for IPNV by Ag-ELISA Kit (Test Line Ltd., Brno, Czech Republic). The experiment was terminated 40 days after introduction of shedder fish. Mortality at the end of the study was defined as endpoint. Statistical analysis was performed using Fisher’s exact test. The relative percent survival (RPS) was calculated by: RPS = (1-cumulative mortality of vaccinated group/cumulative mortality of control group saline) × 100.

## 3. Results

### 3.1. Construction of SAV Replicon Vectors

**Figure 2:**
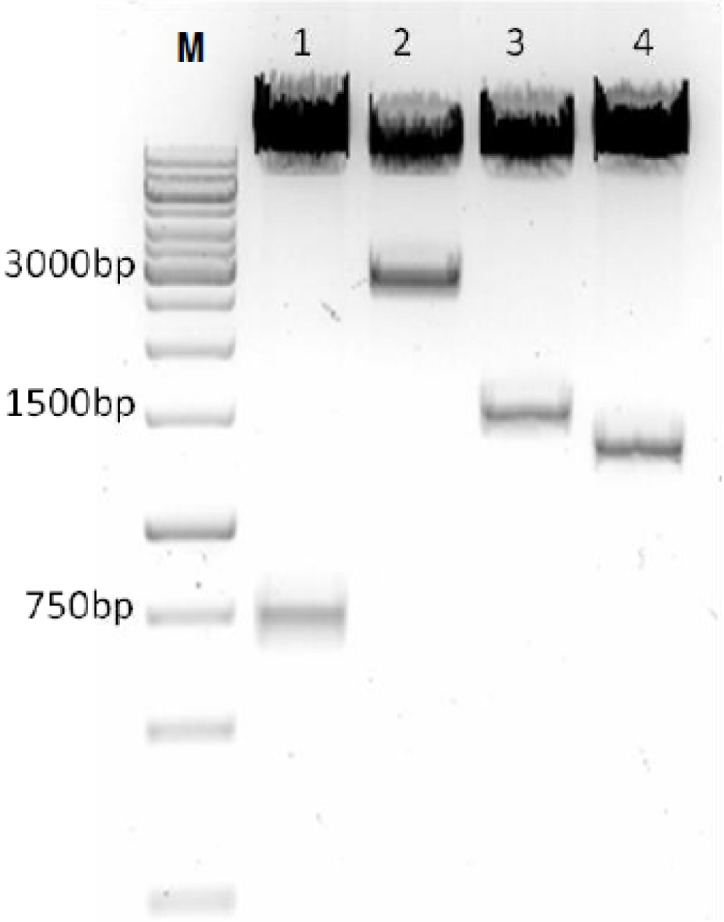
Restiction enzymes analysis of replicon constructs. Each plasmid was digested with *Age1* and *Asc1* restriction sites and analyzed on 1% agarose gel. The size of the products; Lane 1: pSAV/EGFP; lane 2: pSAV/PP; lane 3: pSAV/pVP2 and lane 4: pSAV/VP2; M: Marker (1 kb).

The inserts for pSAV/PP, pSAV/pVP2 and pSAV/VP2 were verified by restriction enzyme analyses (Figure 2), and nucleotide sequencing showed that the introduced mutations were present at positions 18, 218, 784 and 796.

### 3.2. Expression of Recombinant IPNV Proteins in Cell Culture

**Figure 3:**
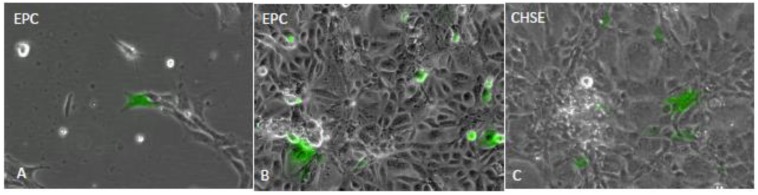
Evaluation of SAV-based replicon expression in EPC and CHSE cells. (**A**) EPC cells transfected with pSAV/EGFP; (**B**) EPC cells transfected with pMAX/EGFP (**C**) CHSE-214 cells transfected with pSAV/EGFP. Pictures were captured by fluorescence microscope at 20× magnification at 48 h post transfection and combined with phase contrast.

**Figure 4:**
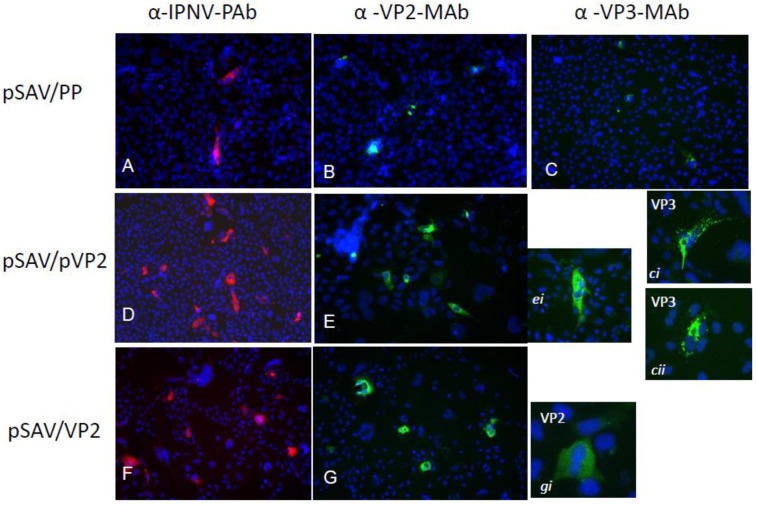
IPNV proteins expression in CHSE-214 cells transfected with pSAV/PP (**A**–**C**); pSAV-pVP2 (**D**) and (**E**); and pSAV-VP2 (**F**) and (**G**); (**A**,**D**,**F**) were immunostained with PAb anti-IPNV; (**B**,**E**,**G**), and close up pictures ei and gi were stained with MAb anti-VP2; (**C**) and close up pictures ci and cii were stained with MAb anti-VP3. Nuclei were counterstained with Hoescht 3334 (blue). Pictures were captured 6 days post transfection at 20× magnification. Secondary antibodies were conjugated with Alexa Fluor 594 (red) and Alexa Fluor 488 (green).

**Figure 5:**
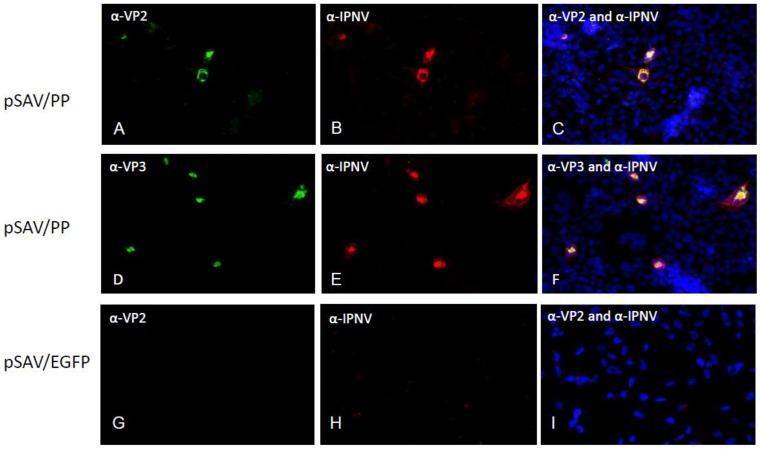
Co-immunostaining of pSAV constructs pSAV/PP and pSAV/EGFP transfected in CHSE-214 cells. (**A**–**C**) were immunostained with MAb anti-VP2 and PAb anti-IPNV; (**D**–**F**) were immunostained with MAb anti-VP3 and PAb anti-IPNV; (**G**–**I**) were pSAV/EGFP transfected (negative control). Nuclei were counterstained with Hoescht 3334 (blue). Pictures were captured 4 days post transfection at 20× magnification. Secondary antibodies were conjugated with Alexa Fluor 594 (red) and Alexa Fluor 488 (green).

Few cells were positive in EPC cultures transfected with pSAV/EGFP (control) (Figure 3A), while many cells were positive in EPC and CHSE (Figure 3B, C) transfected with pMAX/EGFP and pSAV/EGFP, respectively. Similarly, the pSAV/PP, pSAV/pVP2 and pSAV/VP2 showed all higher expression of IPNV proteins in CHSE-214 cells than in EPC cells (data not shown). In CHSE-214 cells maximum expression was observed at 6 dpt. The number of pSAV/pVP2 and pSAV/VP2 positive cells was higher than for pSAV/PP (Figure 4). The expression of VP3 in pSAV/PP transfected cells increased from 4 to 6 dpt, but not visible from 8 dpt and onwards (data not shown). The expression of pVP2 and VP2 pSAV/pVP2 and pSAV/VP2 in transfected cells showed diffuse fluorescence throughout the cytoplasm, excluded from the nucleus (Figure 4 *ei, gi*). A granulated staining pattern was observed for the VP3 expression in pSAV/PP transfected cells (Figure 4 ci, cii). Double staining using PAb anti-IPNV and MAb anti-VP3, or PAb anti-IPNV and MAb anti-VP2, indicated that the polyprotein was successfully translated in pSAV/PP transfected cells as VP2 (Figure 5A–C) and VP3 (Figure 5D–F) staining were found to co-localize PAb-IPNV staining. No staining was observed in the CHSE-214 cells transfected with pSAV/EGFP (Figure 5G–I).

### 3.3. Western Blot

**Figure 6:**
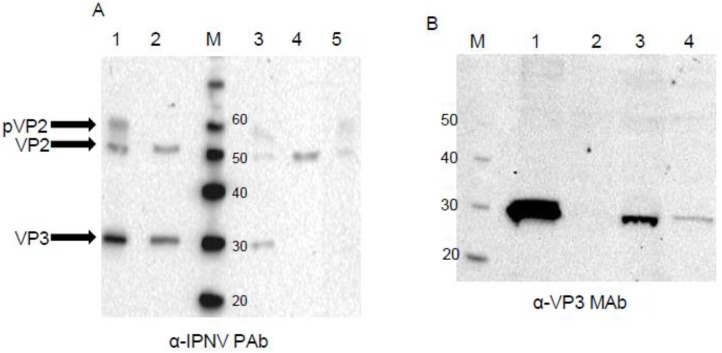
Western blots of CHSE-214 cells transfected with pSAV constructs, IPNV infected CHSE-214 cells lysates and purified IPNV particles. (**A**) Stained with PAb α-IPNV at 4 days post transfected. Lane 1: IPNV infected CHSE-214; lane 2 (positive controls): purified IPNV; M: marker; lane 3: pSAV/PP; lane 4: pSAV/VP2; lane 5: pSAV/pVP2; (**B**) Stained with MAb α-VP3 at 6 days post transfected. Lane 1: purified IPNV (positive controls); lane 2: pSAV/EGFP-transfected (negative control); lane 3: pSAV/PP, culture medium; lane 4: pSAV/PP, cell pellet.

Expression of IPNV proteins after transfection of the different pSAV constructs in CHSE-214 cells was also evaluated by Western blotting. Lysates from IPNV infected CHSE-214 cultures and cesium chloride gradient purified IPNV particles were used as positive controls, and accordingly, pVP2 was present in lysates but not in purified virus (Figure 6A, Lanes 1–2). In pSAV/PP transfected CHSE-214 cell fraction complete polyprotein was not observed, indicating co-translational cleavage in CHSE-214, but pVP2 (faint), VP2 and VP3 were present at 4 dpt (Figure 6A, Lane 3). In pSAV/VP2 only VP2 were seen (Figure 6A, Lane 4), while in pSAV/pVP2 transfected cells both pVP2 (faint) and VP2 were present (Figure 6A, Lane 5).

VP3 was not regularly seen in pSAV/PP-transfected cells at 4 dpt, and at 6 dpt VP3 was more strongly stained from the cell culture medium than from the cell fraction (Figure 6B, Lanes 3–4).

### 3.4. Vaccination Trial

**Figure 7:**
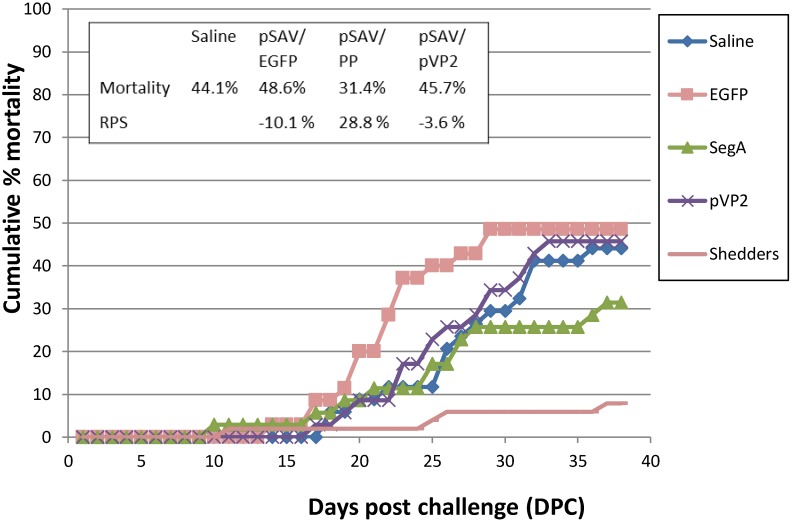
Percentage cumulative mortality of Atlantic salmon smolts in IPN immunization and challenge trial. Control groups (PBS and EGFP) and immunized groups (pSAV/PP and pSAV/pVP2). IPNV injected shedders fish (N = 53) were introduced at Day 0. Cumulative mortality and relative percent survival (RPS) was calculated at termination of challenge.

The replicons pSAV/PP and pSAV/pVP2 were used for immunization of Atlantic salmon smolts in a challenge trial. The fish in the control groups, *i.e.*, injected with PBS and pSAV/EGFP, had a cumulative mortality of 44.1% and 48.6%, respectively. Mortality in the pSAV/PP group started on Day 10 after introduction of shedder fish, and in the other groups on Days 14–17. The mortality rate was slowing down in pSAV/PP group but increased exponentially in PBS, pSAV/EGFP and pSAV/pVP2 injected groups. The mortality patterns of the PBS, pSAV/EGFP and pSAV/pVP2 injected groups closely followed each other (Figure 7). The level of IPNV in head kidneys from 20 dead fish showed that levels of IPNV in the dead fish were highly variable (results not shown). At the end of the challenge trial, the fish in the pSAV/PP group showed a cumulative mortality of 31.4% and RPS of 28.8%, with no significant difference in the cumulative mortality from the PBS group (Figure 7). Bacterial examination of head-kidney samples from dead fish demonstrated the presence of a mixed flora in several individuals.

## 4. Discussion

In this study SAV replicon constructs expressing IPNV proteins were investigated for expression in fish cells and for immunization against IPN. Both the versatility of the SAV replication machinery [31] and the use of the SAV-replicon as an efficacious immunization-vector in aquaculture have shown that this strategy is promising [34,35]. The *in vitro* expression studies demonstrated that IPN proteins were highly expressed in CHSE-214 cells after transfection of the replicon constructs. After transfection of EPC cells with the control pSAV/EGFP less than 1% of the cells were positive, indicating a significant difference in expression efficiency between the cell lines. Transfection of the EPC cell line is in general considered as efficient [37]. The SAV-based replicon has the ability to express in wide range of fish and mammalian cell lines, at a wide temperature range, but with variable levels [31]. The EPC and CHSE-214 cell lines have cyprinid and salmonid origins, respectively, and both are susceptible for many fish viruses. Although EPC lines contaminated with fathead minnow cells have been spread [38], the EPC line that was used was verified as cyprinid after amplification and sequencing of the β-actin gene [37]. However, the EPC cell line is not susceptible for the salmonid viruses infectious salmon anemia virus or SAV [39], indicating that the inhibition of expression of IPNV proteins by the SAV replicon in this cell line was caused by cellular factors.

In immunofluorescence staining, but not in WB, VP3 was strongly stained on 4 dpt, indicating a higher sensitivity for the immunofluorescence assay. In a previous study, the pSAV/EGFP was highly detected on day 4 post transfection in CHSE-214 cells, suggesting the optimum function of SAV based replicon system after 4 dpt in delivering the GOI [31]. During SAV replication in the salmonid TO cell line, the subgenomic transcripts peaked at 4 dpi and then declined [40].

By Western blotting the polyprotein of segment A was found to be proteolytically cleaved into pVP2, VP2, and VP3 on 6 dpt. The VP3, however, was not detected in WB at 8 dpt and onwards, while VP2 was consistently found on 4–8 dpt. VP3 is known to cause apoptosis in infected cells [41]. The presence of pVP2 and VP2 and lack of VP3 in pSAV/PP transfected cells from 8 dpt could indicate selective degradation of VP3, or that its stability is dependent on interaction with dsRNA and VP1, which are natural constituents of IPNV infected cells [13,14].

Ideally, high mortality in unvaccinated fish groups is needed to demonstrate protection by vaccine candidates. Challenge experiments for IPN in Atlantic salmon smolts with reproducible results have been difficult to develop, and it is difficult to obtain consistent IPN mortality in smolts. Mortality in control groups is dependent upon genetic variation of host and virus, age and stocking density of host, and environmental factors [23,42,43]. In IPN challenge experiments higher mortalities in cohabitation groups than in IPNV injected groups are common [44], as we also observed in the present challenge. The mortality in the present study was below 10% in the shedder group and the load of IPNV in head kidneys was highly variable, indicating that the IPNV shedding was low. In addition, a mixed bacterial flora was present post mortem in the head kidney in most individuals. Hence, the trial was considered inconclusive and results of the vaccination trial could only be indicatively assessed. No protection was achieved by pVP2 or VP2 expressing replicons, while the pSAV/PP polyprotein expressing replicon induced a protection that was similar to protection achieved by oil adjuvanted virus antigen vaccine (results not shown). In DNA vaccines expression of the polyprotein either alone or in combination with VP2 protein conferred the highest protection towards IPN [23,45]. In a previous cohabitation challenge of Atlantic salmon smolts, where 33% cumulative mortality was achieved, a RPS of 80% was found after injection of a plasmid expressing the polyprotein, while no protection was achieved for plasmids expressing VP2, parts of VP2 or VP3 [23]. Similarly, in an injection trial in rainbow trout a protective effect of polyprotein expressing plasmid in form of decreased viral load *in vivo* was found [45].

The lack of protective effect by pSAV/pVP2 in the current trial is in line with previous results [23], but still puzzling due to the assumed importance of VP2 in induction of protection; *i.e.*, IPNV-neutralizing MAbs are directed against VP2 [46,47,48,49], the principal antigenic sites, as well as virulence and cell adaptation determinants are present on the VP2 spikes [12,50,51], and 80% RPS was achieved in rainbow trout fry receiving an oral VP2 vaccine [52]. It has been shown that VP3 co-localizes exclusively with the pVP2, and that the interaction between VP3 and pVP2 is important for the particle assembly [53], indicating that VP3 presence is necessary for correct presentation of VP2 epitopes.

Alphavirus replicons have previously been shown to induce stronger immune response than conventional DNA vectors [54]. SAV replicon expressing ISAV hemagglutinin-esterase (HE) induce efficient protection of Atlantic salmon against ISAV challenge [35]. The cytotoxic shutdown of transcription in SAV infected cells is caused by the structural viral capsid protein [55]. The capsid protein is not a part of the replicon and thus the replicon itself is not toxic to the cells, which ensures expression of long duration, as seen by the sustained presence of the expression intermediate dsRNA [31]. dsRNA is a strong inducer of innate immune response and the IFN-, and Mx responses were significantly induced already 6 h and 1 day post vaccination in a SAV-replicon vaccination trial of Atlantic salmon [35].

The use of selective breeding using DNA markers linked to quantitative trait loci (QTL) affecting IPN resistance in Atlantic salmon has recently been found to be an efficient mean to achieve protection [56], both in sea water as well as fresh water [57]. However, the potential rapid evolution of RNA viruses, such as IPNV, could make selective breeding vulnerable for escaped mutant viruses. Therefore, development of efficient vaccines would form an additional safeguard against the disease.
